# Distress and burnout among psychiatrists during the COVID-19 pandemic

**DOI:** 10.1192/j.eurpsy.2021.826

**Published:** 2021-08-13

**Authors:** N. Bassetti, S. Parente, P. Topa, N. Brondino, S. Damiani, P. Politi, M. Olivola

**Affiliations:** Department Of Brain And Behavioral Sciences, University of Pavia, Pavia, Italy

**Keywords:** COVID-19, distress, psychiatrists, burnout

## Abstract

**Introduction:**

COVID-19 is an infectious disease caused by SARS-CoV-2. The WHO on March 11, 2020, has declared the novel coronavirus outbreak a global pandemic. Several studies found an association between the COVID-19 pandemic and psychiatric symptoms, such as distress, anxiety, fear of infection, depression and insomnia in the general population. Therefore, psychiatrists have been professionally overloaded, trying to manage the psychosocial impact of the pandemic and suffering its effects in person.

**Objectives:**

To evaluate the disease perceptions, distress and burnout among psychiatrists from the Department of Mental Health and Addictions of Pavia in three different times, which correspond to the three main phases of the pandemic management in Italy: T0 is the first peak of the infections and the lock-down, from March to June; T1 is the reduction of the infections and the reopening, from June to October; T2 is the second wave of infections with a new progressive closure, the current one.

**Methods:**

We used three questionnaires: the BIPQ (Brief Illness Perception Questionnaire), the PSS-10 (Perceived Stress Scale-10), the PED (Profile of emotional distress). We also used a survey (6 items) in T0, T1 and T2 to evaluate exposure, perception, quality of life and burnout.

**Results:**

**Figure:**
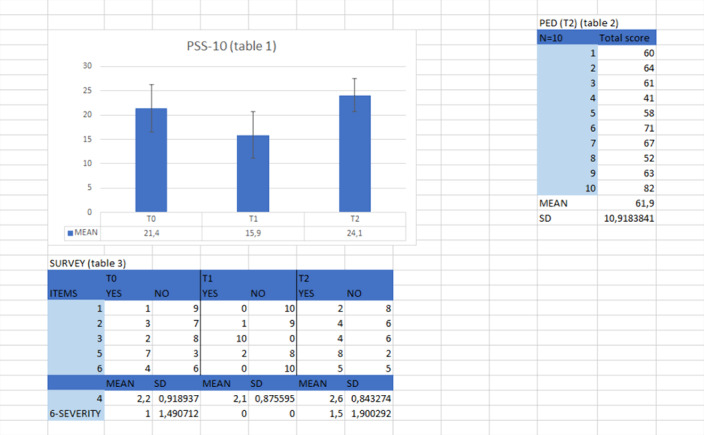

table 1,2,3. BIPQ: no one was exposed.

**Conclusions:**

The increase of individual, who seeking help for mental health, impact on the perception of stress and on the emotional distress, even though psychiatrists have an adequate perception of COVID-19.

